# Higher-order repeat structure in alpha satellite DNA occurs in New World monkeys and is not confined to hominoids

**DOI:** 10.1038/srep10315

**Published:** 2015-05-14

**Authors:** Penporn Sujiwattanarat, Watcharaporn Thapana, Kornsorn Srikulnath, Yuriko Hirai, Hirohisa Hirai, Akihiko Koga

**Affiliations:** 1 Primate Research Institute, Kyoto University, Inuyama City 484-8506, Japan; 2 Faculty of Science, Kasetsart University, Bangkok 10900, Thailand

## Abstract

Centromeres usually contain large amounts of tandem repeat DNA. Alpha satellite DNA (AS) is the most abundant tandem repeat DNA found in the centromeres of simian primates. The AS of humans contains sequences organized into higher-order repeat (HOR) structures, which are tandem arrays of larger repeat units consisting of multiple basic repeat units. HOR-carrying AS also occurs in other hominoids, but results reported to date for phylogenetically more remote taxa have been negative. Here we show direct evidence for clear HOR structures in AS of the owl monkey and common marmoset. These monkeys are New World monkey species that are located phylogenetically outside of hominoids. It is currently postulated that the presence of HOR structures in AS is unique to hominoids. Our results suggest that this view must be modified. A plausible explanation is that generation of HOR structures is a general event that occurs occasionally or frequently in primate centromeres, and that, in humans, HOR-carrying AS became predominant in the central region of the centromere. It is often difficult to assemble sequence reads of tandem repeat DNAs into accurate contig sequences; our careful sequencing strategy allowed us to overcome this problem.

The centromere is part of a chromosome essential for correct chromosome segregation during cell division, serving as the point to which the spindle fiber attaches via the kinetochore. Centromeres of higher eukaryotes generally contain large amounts of tandem repeat DNA. Alpha satellite DNA (AS) is the most abundant tandem repeat DNA of primate centromeres^1^,^2^, although this may not be true of suborder Strepsirrhini, one member of which (the aye-aye, *Daubentonia madagascariensis*) is known to carry other unrelated tandem repeat DNA as main components of its centromeres^3^. Strepsirrhini is a taxon that diverged from other groups in an early stage of the primate evolution. The length of the repeat units of AS is approximately 170 bp in parvorder Catarrhini, which includes hominoids (superfamily Hominoidea; humans, great apes, and small apes) and Old World monkeys (family Cercopithecidae; macaques, baboons, and related monkeys found in Africa and Asia)^4^,^5^,^6^. In New World monkeys (parvorder Platyrrhini; monkeys inhabiting Central and South America), the length of the repeat units is approximately 340 bp^6^,^7^. Sequence analyses have suggested that this difference is due to an ancient dimeric structure of AS of New World monkeys^6^. The parvorders Catarrhini and Platyrrhini constitute the infraorder Simiiformes (simian primates).

The AS of humans, which has been extensively studied for its structural features, is known to contain sequences organized into higher-order repeat (HOR) structures, which are tandem arrays of larger repeat units that consist of multiple basic repeat units^8^,^9^. The larger repeat units that have so far been identified include those comprising 2, 4, 5, 6, 8, 11 and 13 basic repeat units^9^,^10^,^11^,^12^,^13^,^14^,^15^. The HOR structure also occurs in other hominoid species^2^,^5^, including gibbons^16^,^17^, but has not been reported in Old World monkeys or New World monkeys. For this reason, it is currently postulated that the HOR structure of AS is a unique attribute of hominoids^6^,^16^. In the present study, we asked whether the HOR structure occurs in a taxon that is located phylogenetically outside of hominoids. We raised this question because the repetitive DNA sequences that constitute aye-aye centromeres (called DMA1 and DMA2) exhibit the HOR structure^3^. The aye-aye is phylogenetically more remote from hominoids than Old World monkeys or New World monkeys are. It is possible that the HOR structure has not been identified in Old World monkeys or New World monkeys simply because the experimental methods used to date lack sufficient detection power or because the data currently available in sequence databases are of insufficient quality.

In our previous studies^16^,^17^, we identified the HOR structure in AS of gibbons by obtaining accurate, long sequences of AS-carrying genomic DNA clones. Our strategy consisted of two steps. First, we selected AS-carrying clones that appeared to contain the HOR structure. Second, we sequenced a long region within the identified candidate clone. By inserting a bacterial transposon into the candidate clone at various positions, we collected two kinds of information: the position of transposon insertion, and the sequences of the transposon-flanking regions. Using the former positional information to collate the latter sequence data, we constructed accurate contig sequences of the long regions. In the present study, we further improved our method and applied it to AS of Azara’s owl monkey (*Aotus azarae*) and the common marmoset (*Callithrix jacchus*) and obtained clear evidence of the HOR structure outside the hominoid lineage.

## Results

### Screening of genomic library for AS-carrying clones

In our previous study^18^, we identified two types of AS in the owl monkey, which we named OwlAlp1 (185-bp repeat units) and OwlAlp2 (344-bp repeat units). Our cloning method employed genomic hybridization against fosmid clones randomly selected from an owl monkey genomic library. In the present study, we conducted another round of library screening using the same method. Starting with 384 clones (contained in four 96-well plates) randomly selected from the genomic library, we picked 24 clones that exhibitied the highest levels of signal intensities. One end (>750 bp) of the insert fragments of the 24 clones was sequenced with a primer that represented one terminal region of the pCC1FOS vector. Six of the 24 clones were found to contain tandem repeat structures showing sequence identities of >90% with the consensus sequence of OwlAlp1, and another six clones showed sequence identities of >90% to the consensus sequence of OwlAlp2^18^. Of the remaining 12 clones, nine were found to contain OwlRep, another tandem repeat DNA present in large amounts in the owl monkey genome^19^.

Using the same method, from the marmoset library, we collected 24 clones that were presumed to carry tandem repeat DNA. We examined the terminal regions of six of these 24 clones, and all exhibited a >90% sequence identity with the consensus sequence of the marmoset AS^6^.

### Detection of an HOR sign by partial sequencing

We delivered a modified Tn5 transposon to the six OwlAlp2 and six marmoset AS clones, and collected those carrying the transposon in their insert portions. For each original clone, we sequenced three transposon-carrying clones for transposon-flanking regions. The three clones were selected so that their insertion points would be apart from one another by >3 kb in order to ensure no overlap among their sequence reads. We then compared the sequence data with a dot matrix analysis for a sign of the HOR structure. The logic underlying this test has been described in our previous report^11^. Briefly, if a line spanning more than two repeat units on a dot matrix is observed, it can be regarded as an HOR sign.

**Figure 1:**
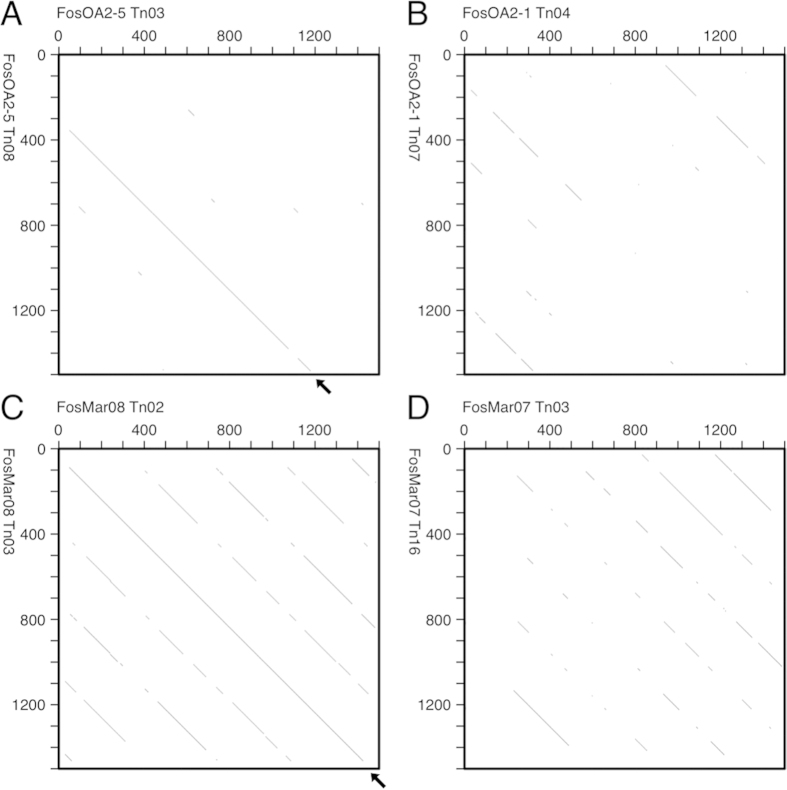
Dot matrix analysis of sequence reads for a sign of the HOR structure. The criterion in the analysis was that a 49- or 50-nucleotide match should exist over a window of 50 nucleotides. A line spanning more than two basic repeat units (approximately 680 bp), if found, is regarded as a sign for an HOR structure. Examples of the results are shown. **A**. Fosmid clone FosOA2-5 of owl monkey that showed a sign. **B**. FosOA2-1 of owl monkey that was negative. **C**. FosMar08 of marmoset that showed a sign. **D**. FosMar07 of marmoset that was negative.

We found signs of the HOR structure in two of the six owl monkey clones (FosOA2-2 and FosOA2-5) and one of the six marmoset clones (FosMar08), as shown in Fig. 1 (negative cases are also shown).

### Construction of long contig sequences

We conducted further experiments, using FosOA2-5 and FosMar08, to obtain long contig sequences of the AS. The insert fragments of these clones were tandem repeat DNA and, in addition, were considered to contain identical sequence blocks at multiple locations because of the anticipated HOR structure. For this reason, we could not rely on the shotgun or primer-walking sequencing strategies. We used the same strategy, with a small modification, as that employed in our previous study^17^, in which multiple sequence reads were collected together with the location data of the sequence reads and they were assembled into a contig sequence based on their location. The modification that we made in the present study was to collect fosmid clones that carried the transposon at various locations and sequence these fosmds to obtain the transposon-flanking regions. In our previous method, an insert fragment was transferred once to a plasmid and a series of nested deletion clones were prepared with restriction endonucleases and an exonuclease. This modification was effective in reducing the number of clones to be sequenced because the length of each sequence stretch was doubled by reading it in both directions from a single point. The modification was also useful for reducing the possibility of artificial sequence rearrangements by removing the subcloning step^20^.

For the FosOA2-5 clone, we obtained a total of 18 partially overlapping sequence reads and assembled them into a 10829-bp contig sequence. For the FosMar08 clone, we collected 24 sequence reads, which were assembled into a 13124-bp sequence. We deposited these sequences in GenBank under the accession numbers LC002884 and LC030305, respectively.

### Evidence for HOR structure from the contig sequences

**Figure 2:**
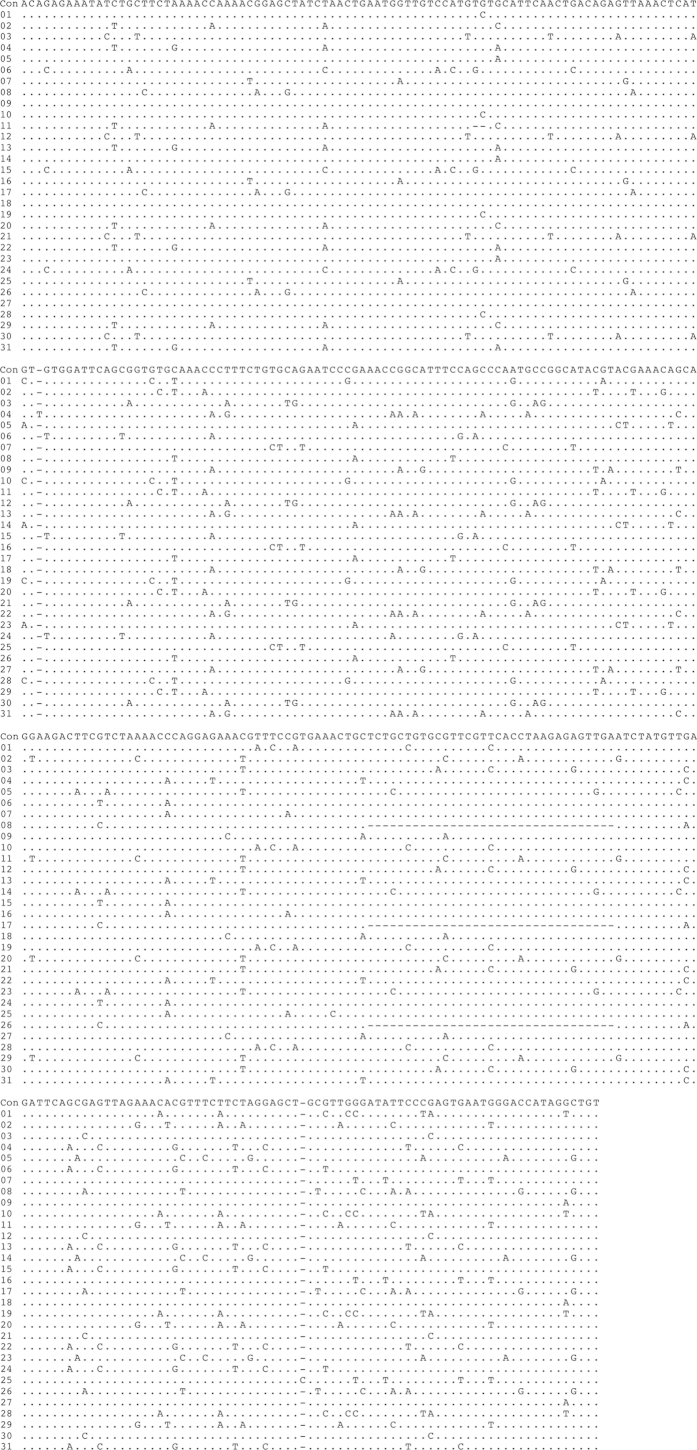
Alignment of the nucleotide sequences of the 31 basic repeat units in the FosOA2-5 clone. The alignment was performed with the MEGA6 program^24^ under default settings. *Con* indicates the consensus sequence that was assembled by collecting the most common nucleotides at the respective sites. Nucleotide sites occupied by the base in the consensus sequence are indicated by dots. Nucleotide sites containing different bases are shown as the respective bases observed. The minus symbol indicates the absence of a nucleotide at that position.

**Figure 3:**
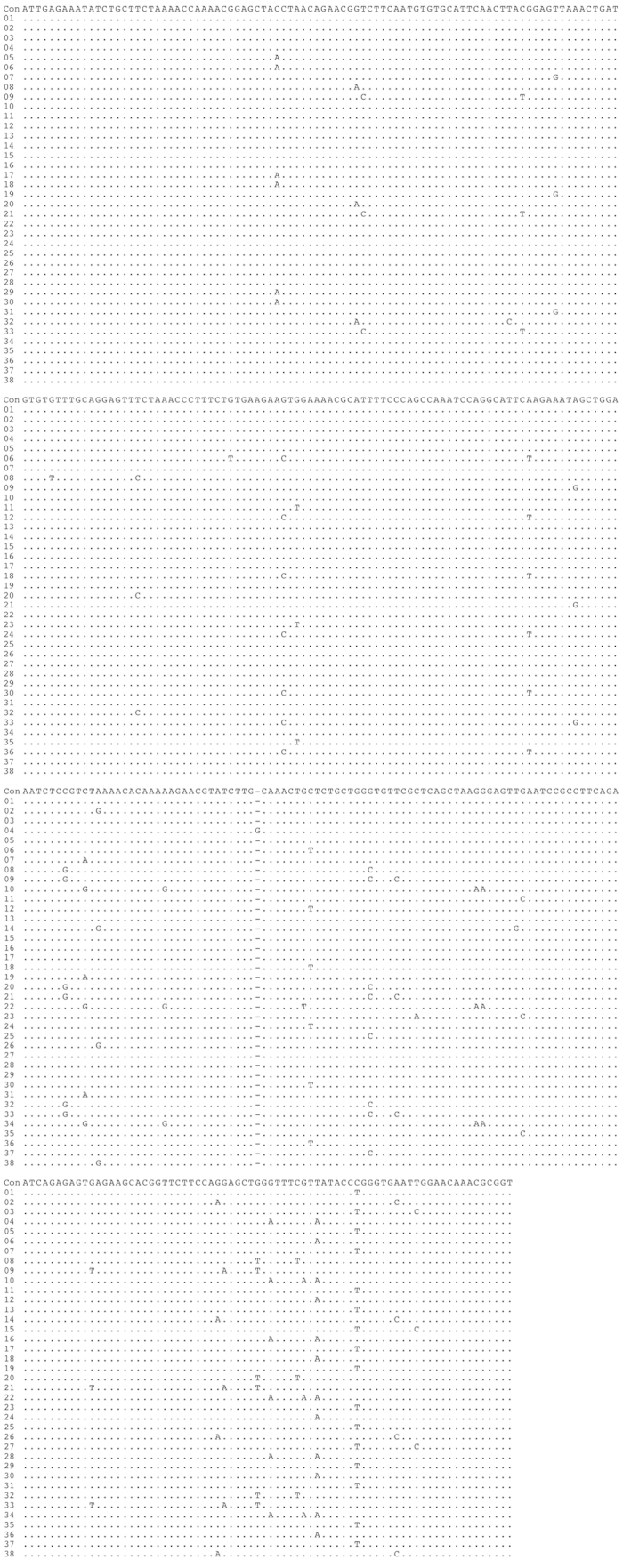
Alignment of the nucleotide sequences of the 38 basic repeat units in the FosMar08 clone. The sequence data obtained from the FosMar08 clone were treated by the same methods as those described in the legend to Fig. 2.

**Figure 4:**
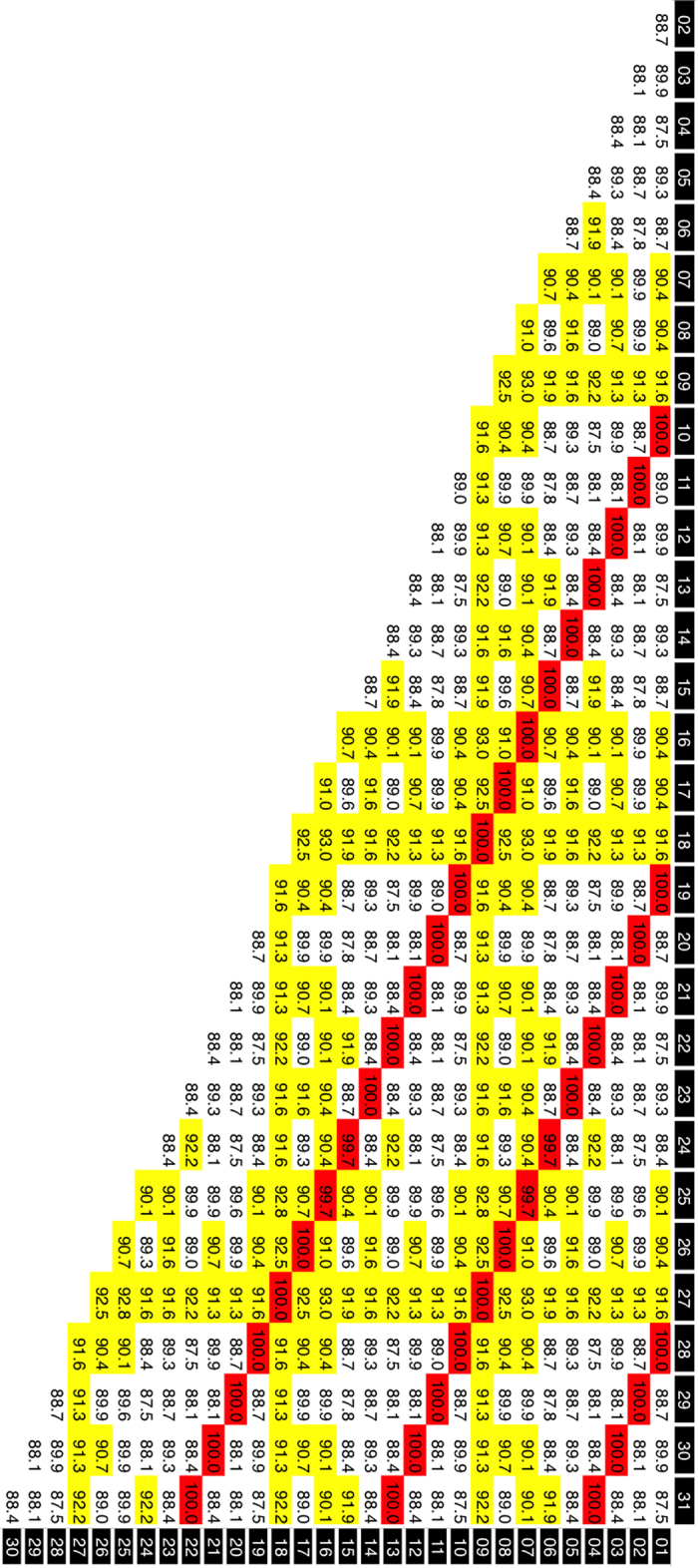
Pairwise comparisons of basic repeat unit sequences in the FosOA2-5 clone. The comparisons were made with the MEGA6 program^24^ under default settings. The horizontal and vertical axes of each matrix represent a one-dimensional array of the basic repeat units. Each cell contains the nucleotide identity of the corresponding pair. The 31 basic repeat units in the FosOA2-5 clone. Yellow cells, nucleotide identities of 90.0–95.0; red cells, >95.0%.

**Figure 5:**
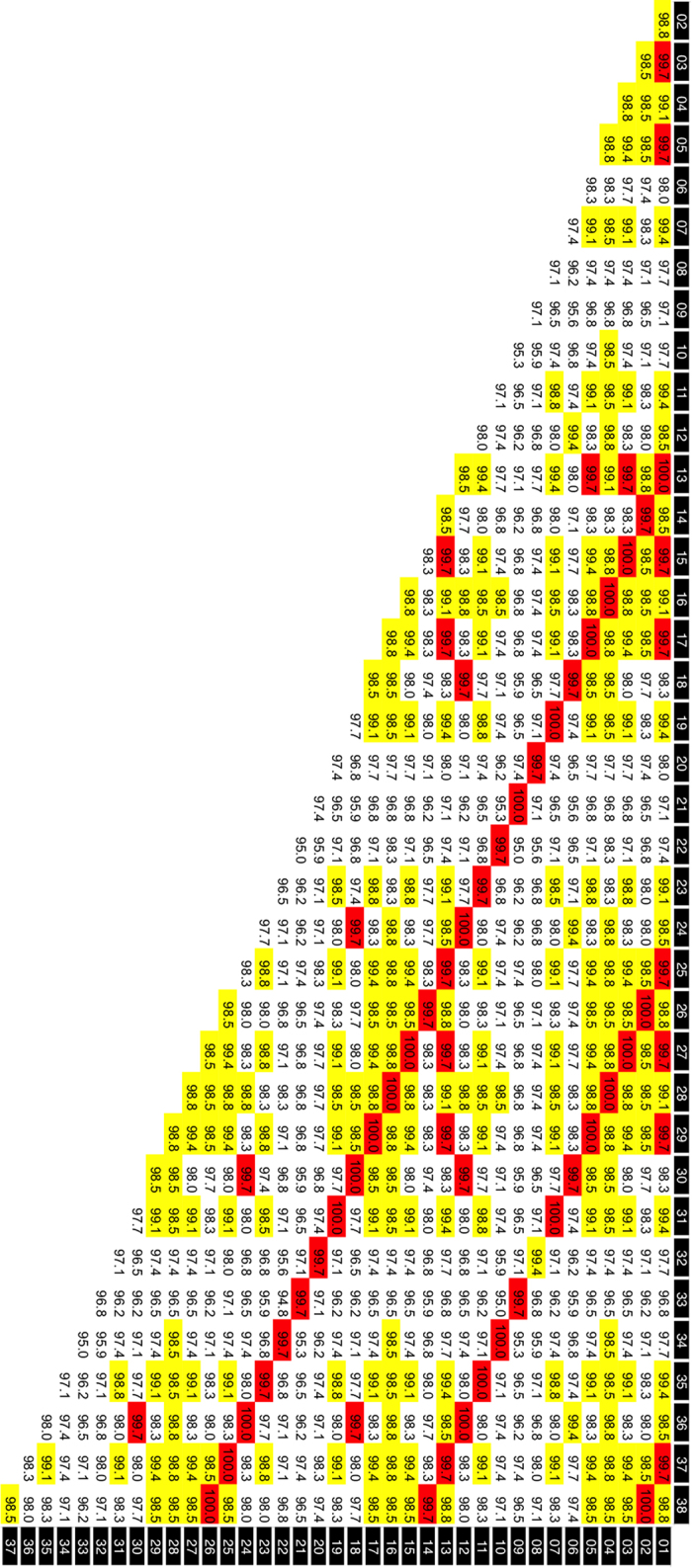
Pairwise comparisons of basic repeat unit sequences in the FosMar08 clone. The sequence data obtained from the FosMar08 clone were treated by the same methods as those described in the legend to Fig. 4, except for the coloring criteria: yellow cells, nucleotide identities of 98.5–99.5%; red cells, >99.5%.

The contig sequences derived from the FosOA2-2 and FosMar08 clones contained 31 and 38 basic repeat units, respectively, for which the same boundaries of the repeat units as those defined in our previous study^18^ were used. The alignment of these repeat units (Figs. 2 and 3) suggested the presence of the HOR structure, showing repetitions of several similar patterns along the sequence. Pairwise comparisons of the sequence identities between the repeat units (Figs. 4 and 5) provided clear evidence for the HOR structure: mutually parallel lines that consisted totally or mostly of red cells appeared against a background comprised mostly of yellow and white cells. The average pairwise identities among the basic repeat units are shown in Table 1. The distance (number of cells along the axis) between the parallel lines in Figs. 4 and 5 corresponds to the size of the larger repeat units, which was nine and 12 basic repeat units in FosOA2-2 and FosMar08, respectively.

## Discussion

The HOR structure in AS has been found in humans and other hominoid species, but has not been reported in phylogenetically more remote primate taxa, such as Old World monkeys or New World monkeys. For this reason, it is currently postulated that the HOR structure is a unique attribute of hominoids. In the present study, we observed clear HOR structures in AS of the owl monkey and marmoset, which are New World monkey species. Our results suggested that the HOR structure occurs in AS of a wide range of simian primates. We undertook this study to identify the HOR structure in AS of New World monkeys partly because the HOR structure has already been identified in the centromere-region repetitive DNA (DMA1 and DMA2) of the aye-aye^3^, a species phylogenetically located more remote from hominoids than New Workd monkeys. This raised the possibility that the HOR structure is common in AS of simian primates, and is not confined to hominoids. It now appears possible that the generation of an HOR structure is a general event occurring occasionally or frequently in centromeres of all simian primates and that, in humans, HOR-carrying AS became predominant in the central region of the centromere by being associated with a significant centromere function, changes in the turnover mechanisms of AS, and/or other unknown mechanisms.

Detection of an HOR structure in AS of Old World monkeys and New World monkeys has been attempted using various methods, including restriction enzyme analysis of genomic DNA^4^,^5^,^7^,^21^, and computational analysis of shotgun sequence databases^5^,^6^. To our knowledge, however, no results have been positive. The restriction enzyme method has the potential to succeed only when the larger repeat units carry the recognition sites of the enzymes used. For the computational analyses tried to date, the original data to be analyzed (sequence reads generated by current sequencing machines) may not be long enough for detection of an HOR structure. For the marmoset AS in particular, the high nucleotide identity among repeat units may pose another obstacle to detection of an HOR structure. In the human AS, the average identities among basic repeat units located at identical and nonidentical positions (in different larger repeat units) have been reported to be 95–99% and 70–90%, respectively^2^,^5^,^9^. The HOR structures of gibbon AS that we identified in our previous studies exhibited values comparable with these^16^,^17^. In the marmoset AS, however, the nucleotide identities were 99.9% and 97.9%, respectively. The overall identity was as high as 98.0%.

The method we used in the present study was sufficiently powerful to detect the HOR structure. Some drawbacks of this method are that it requires much manual handling of clones and that it is time-consuming. Clones of repetitive DNA are often degraded easily when maintained or amplified in the host bacteria^20^. The marmoset AS clones were far more fragile than the other repetitive DNAs we have so far analyzed, probably due to their high sequence homogeneity, and they required more careful handling. Despite these drawbacks, our method is, to our knowledge, the only method to provide clear and direct evidence of the HOR structure in AS of New World monkeys.

## Methods

### Ethics statement

All animal experiments in this study were approved by the Animal Care and Use Committee of Kyoto University Primate Research Institute (KUPRI), and were performed in accordance with the Guidelines for Care and Use of Nonhuman Primates (Version 3; June 2010) of KUPRI.

### Azara’s owl monkey, genomic library, and library screening

We used the same *A. azarae* genomic library as that used in our previous study^16^. The essential information is as follows: source of genomic DNA, cultured epithelial cells originating from an adult female; vector, fosmid pCC1FOS that is 8.1 kb in length and carries the chloramphenicol-resistance gene; and insert DNA, 40- to 44-kb fragments produced by mechanical shearing. We screened this library for repetitive sequences by the genomic hybridization technique, of which the strategy and methods have been described in our previous studies^18^,^22^,^23^. We used the AlkPhos Direct Labelling and Detection System (product of GE Healthcare) for hybridization and signal detection. The probe used was owl monkey genomic DNA that had been mechanically sheared to lengths of around 10 kb. The hybridization temperature was 59 °C, at which a medium hybridization stringency was expected.

### Common marmoset

Genomic DNA was extracted from cultured epithelial cells of a male common marmoset (bred at KUPRI; individual identification number 186). The method for the library construction and screening was the same as that for the owl monkey. The probe used for the hybridization was marmoset genomic DNA sheared to lengths of around 10 kb.

### Preparation of fosmid clones carrying transposon insertions

The bacterial transposon Tn5 was used for detection of signs of the HOR structure and sequencing of candidate clones. We induced an *in vitro* transposition reaction and collected secondary fosmids that carried the transposon on the original fosmid. The methods for these processes have been described in full previously, with schematic illustrations^17^. The Tn5 transposon was modified in advance to carry the kanamycin-resistance gene for the selection of insertion-carrying fosmids and some restriction enzyme sites for determining Tn5 insertion points by restriction mapping.

### Sequencing of terminal regions and transposon-flanking regions

Fosmid DNAs were sequenced by Sanger’s method with an Applied Biosystems 3730*xl* DNA Analyzer. To obtain sequences of the terminal regions of the insert fragment, we used primers that represented nucleotide blocks encompassing the insertion site of the pCC1FOS vector (nucleotides 276–305 of GenBank file EU140751). Each sequencing assay provided a sequence read of >1000 bp, from which we used the first 750 bp as an accurate sequence data. For the sequence data of the transposon-flanking regions, we used primers that represented the left and right terminal regions of the modified transposon and were oriented outwards (5’-GAATTTTGAATTCGGTACCATGCGGCCGCT-3’ and 5’-TGAGCGGCCGCTAAAGCTTCTAGACCAACA-5’). By combining the pair of sequence reads at the transposon insertion breakpoint, we could obtain a sequence stretch of >2000 bp. We used its internal 1500-bp portion, 750 bp each from the junction point, as accurate sequence data.

The fosmid vector pCC1FOS is present as a single copy in bacterial cells because it is controlled by the replication origin *ori*2. This vector carries another replication origin, *ori*V, that leads to multiple copies by exposing bacterial cells to an induction reagent. The induction of multiple copies is usually applied for sequencing purposes. We, however, did not include multiple copy induction in any part of our experiments. The OwlAlp2 sequence is fragile when carried by a cloning vector, especially a multiple-copy vector^20^, and the FosMar08 sequence is even more fragile. To obtain accurate sequence data by preventing structural changes, we treated the bacterial culture so that the single-copy situation would be maintained. The reduction in the DNA amount that was caused by the no induction treatment was compensated for by increasing the culture volume from 1 ml to 16 ml per sequencing reaction.

The temperature of bacterium culturing is another significant factor for avoiding structural changes. The FosOA2-5 and FosMar08 clones were highly fragile when cultured at 37 °C, but stable when cultured at 25 °C and 22 °C, respectivel. We conducted bacterium culturing at these temperatures. Because the bacterium growth was slow at these low temperatures, the culturing time was extended, from regular overnight culturing, to 48 h.

## Author Contributions

A. K. conceived and designed the experiments. P. S., W. T., and A. K. performed molecular biology experiments. Y. H. and H. H. conducted cytology experiments. K. S., H. H. and A. K. carried out data analysis.

## Additional Information

**How to cite this article**: Sujiwattanarat, P. *et al.* Higher-order repeat structure in alpha satellite DNA occurs in New World monkeys and is not confined to hominoids. *Sci. Rep.* **5**, 10315; doi: 10.1038/srep10315 (2015).

## Figures and Tables

**Table 1:** Average pairwise identities.

**Clone**	**FosOA2-5**	**FosMar08**
All pairs		
No. of pairs	31 × 30/2 = 465	38 × 37/2 = 703
Average identity	90.7%	98.0%
Pairs at identical positions^a^		
No. of pairs	22 + 13 + 4 = 39	26 + 14 + 2 = 42
Average identity	100%	99.9%
Pairs at nonidentical positions^b^		
No. of pairs	465-39 = 426	703-42 = 661
Average identity	89.8%	97.9%

^a^Pairs of basic repeat units that were located at identical positions in different larger repeat units (pairs on the mutually parallel lines that consist totally or mostly of red cells in Figs. 4 and 5).

^b^Pairs of basic repeat units not bearing the above-mentioned relationship.
